# The influence of physical exercise on achievement motivation among college students: the mediating roles of self-efficacy and life satisfaction

**DOI:** 10.3389/fpsyg.2025.1529829

**Published:** 2025-01-29

**Authors:** Longan Cao, Qianhang Yu, Xin Feng, Lei Wang, Jun Lang

**Affiliations:** ^1^School of Physical Education, Southwest University, Chongqing, China; ^2^Faculty of Sports Science, Ningbo University, Ningbo, China; ^3^Teaching and Research Association, CaiJia Primary, Chongqing, China

**Keywords:** physical exercise, college students, self-efficacy, life satisfaction, achievement motivation

## Abstract

**Background:**

As an inherent cognitive process in the pursuit of progress among college students, achievement motivation has become an indispensable facet of daily life. This study aims to investigate the association between physical exercise and achievement motivation, while also examining the mediating roles played by self-efficacy and life satisfaction.

**Methods:**

This study employed a cross-sectional survey methodology, with a sample of 900 Chinese university students as the research participants. A total of 844 valid questionnaires were collected and analyzed. The participants completed various scales, including measures of sports activity level, self-efficacy, life satisfaction, and achievement motivation. Subsequently, comprehensive descriptive statistical analyses were conducted on the gathered data.

**Results and conclusion:**

A significant positive correlation is present between physical exercise and achievement motivation (r = 0.464, *p* < 0.01), self-efficacy (r = 0.288, *p* < 0.01), and life satisfaction (r = 0.333, *p* < 0.01) among college students. Moreover, achievement motivation demonstrates a positive association with self-efficacy (r = 0.506, *p* < 0.01) and life satisfaction (r = 0.399, *p* < 0.01). Furthermore, the relationship between physical exercise and achievement motivation can be influenced by both self-efficacy and life satisfaction as mediators in our constructed mediation model where the total effect is 0.512 with direct effect of 0.348 and indirect effect of 0.163. The results of the constructed mediating effect model demonstrate that self-efficacy and life satisfaction serve as significant mediators and moderators, effectively enhancing the achievement motivation levels of college students. This study provides novel insights for enhancing intervention strategies to improve levels of achievement motivation not only among Chinese but also global college students.

## 1 Introduction

As the vanguard of society in terms of emerging technologies and innovative ideas, and as highly specialized talents nurtured by the nation, college students’ profound drive for achievement has consistently garnered attention from various sectors (Bowman, 2010). However, in the post-epidemic period, college students are confronted with multifaceted pressures including academic pursuits and career prospects. These diverse factors exert varying degrees of influence on the achievement motivation of college students.

Achievement motivation refers to an individual’s inclination to exert effort in accomplishing valuable tasks and excelling in them, encompassing the pursuit of success and avoidance of failure (Atkinson, 1963). According to achievement motivation theory, individuals with a pronounced need for achievement aspire to complete tasks impeccably and attain higher levels of success (Wang et al., 2019). Previous research has demonstrated that achievement motivation significantly impacts the academic performance (Bjørnebekk et al., 2013), job-related stress (Li et al., 2022), life stress (Karaman and Watson, 2017), and self-expression of college students (Fahmi et al., 2019). To address this issue effectively, it is imperative for individuals to cultivate a proper mindset that fosters increased motivation in order to attain their goals or fulfill personal needs (Brunstein and Schmitt, 2004). Derogatory terms such as “lying flat” and “crispy college students” have become labels for certain behaviors. Underlying this phenomenon is the observation that, when faced with tasks, some college students fail to maintain a proactive and positive attitude, instead adopting a mentality of procrastination or avoidance, indicative of low achievement motivation (Fong et al., 2024). This phenomenon may result in a deficiency of the fighting spirit that should characterize college students in their pursuit of a better life, leading to inertia in future work and a lack of diligence. Consequently, investigating the influencing factors and internal mechanisms underlying achievement motivation among college students holds immense theoretical value and practical significance in nurturing their drive for success while alleviating the burden imposed by both internal and external challenges.

Physical exercise serves as a means of regulating college students’ achievement motivation, involving sports activities of specific intensity, frequency, and duration undertaken with the aim of maintaining health during leisure time (Song, 2001). Based on the theory of embodied cognition, it is assumed that the condition of the body directly affects cognitive and physiological processes, as well as its interaction with the external environment (Ye, 2011). In other words, physical exercise has a direct impact on motivation. For example, Chen’s “Physical Exercise Attitude Scale” consists of 70 items and 8 dimensions (Chen et al., 2006). Although this scale evaluates individuals’ psychological attitudes towards various aspects of sports, it does not provide sufficient clarity regarding college students’ current performance in sports implementation (Li X. N. et al., 2024; Li J. et al., 2024; Zhou et al., 2024). This means there may be some deviation in defining the impact of physical activities on achievement motivation in this study. Therefore, we will use a more appropriate Physical Activity Rating Scale-3 (PARS-3) in subsequent evaluations to make the determination of achievement motivation level more intuitive and evident (Cavicchiolo et al., 2022). Consistent with previous research findings, physical exercise plays a crucial role in the body by effectively reducing stress and generating motivational benefits that stimulate adaptive responses (Brown and Fry, 2013). And positive motivation can promote advancements in sports activities and result in beneficial outcomes encompassing health, aesthetics, enjoyment, skill development, and social interaction (Li X. N. et al., 2024; Li J. et al., 2024). Therefore, it can be inferred that there is a mutually reinforcing relationship between physical exercise and achievement motivation. However, despite schools being widely regarded as ideal environments for promoting sports, enhancing achievement motivation, and improving the mental health of adolescents (Morton et al., 2015), there is a lack of crucial data in the college student population to comprehensively evaluate the effectiveness of physical education programs provided by educational institutions in fostering achievement motivation. Moreover, within the learning environment, approximately 4.5 million students join the ranks of Chinese college students annually. While there is a collective drive for excellence and high societal expectations, this large student population exhibits significant variability in motivation levels. Consequently, the overall motivational impact has not seen substantial improvement (Sun et al., 2023). Consequently, this study takes Chinese university students as the sample and hypothesizes that there is a positive relationship between physical exercise and achievement motivation.

Although physical exercise may directly enhance college students’ levels of motivation for achievement, there is still a lack of comprehensive understanding regarding the potential influence of other factors on the process of promoting achievement motivation through physical exercise. Self-efficacy refers to individuals’ confidence in their ability to utilize their own cognitive resources to successfully complete specific tasks or behaviors (Bandura, 1977). It represents an essential unconscious behavior reflecting an individual’s belief in taking appropriate actions to cope with environmental demands (Sarı and Bayazıt, 2017). For instance, individuals with high general self-efficacy perceive themselves as more capable of performing well (Kriegbaum et al., 2015). Additionally, there is a positive correlation between self-efficacy and achievement motivation. Increasing one’s sense of self-efficacy can facilitate the development of achievement motivation, enabling students to cultivate a desire for autonomy and competence, thereby overcoming obstacles and achieving task proficiency (Bandura and Schunk, 1981; Niemiec and Ryan, 2009). However, certain research findings suggest that students with high self-efficacy may rely excessively on their motivational thinking, resulting in a lack of concentration during learning and detachment from self-confidence (Katz et al., 2011; Bailey et al., 2023). Therefore, based on the individual differences observed in self-efficacy research, this study proposes the hypothesis that “self-efficacy acts as an intermediary factor between physical exercise and motivation for academic achievement” to empirically examine these outcomes.

In the college experience, students are bound to encounter setbacks and challenges that may lead to feelings of discouragement; therefore, it is imperative to enhance their level of achievement motivation. In this context, life satisfaction refers to an individual’s cognitive evaluation of their overall happiness in life (Huebner, 2004), serving as a crucial indicator for assessing personal quality of life and sense of well-being (Diener et al., 1999). Research findings indicate that life satisfaction plays a pivotal role in fostering achievement motivation, as students who exhibit higher levels of life satisfaction across multiple dimensions (e.g., environment, family, school, peers, self) demonstrate increased engagement and concentration while undertaking tasks (Hakimzadeh et al., 2016; Datu and King, 2018). For example, the provision of support from both family members and educators can enhance life satisfaction levels, thereby establishing an enabling learning environment and bolstering the manifestation of achievement motivation (Foster et al., 2017). Moreover, there exists a strong association between participation in sports activities and life satisfaction; regular involvement in physical pursuits has the potential to enhance overall life satisfaction among students (Arslan and Akkas, 2013; Gül and Küçükibiş, 2018). Taking into account the aforementioned perspectives, life satisfaction not only enhances individual motivation for achievement but also potentially acts as a mediating mechanism between physical exercise and achievement motivation. Based on this premise, we propose H3: Life satisfaction serves as an intermediary in the relationship between physical exercise and achievement motivation.

Although existing literature suggests that self-efficacy and life satisfaction independently influence the relationship between physical exercise and achievement motivation, it is important to acknowledge their interconnection. For example, individuals with high self-efficacy are more likely to effectively solve problems, thus enhancing overall happiness in life (Luthans et al., 2007; Coffey and Warren, 2020). Additionally, higher levels of life satisfaction among college students contribute to improved emotional regulation and flexible problem-solving approaches which subsequently mitigate the risks associated with internalizing or externalizing issues (Forrest et al., 2013). Therefore, it is reasonable to propose that engaging in physical exercise can evoke positive emotional experiences while enhancing beliefs in one’s own abilities (self-efficacy), ultimately promoting life satisfaction as a means of fostering optimal achievement motivation for goal attainment (Anderson and Brice, 2011; Pavin Ivanec and Defar, 2023). As stated in the following assumption, self-efficacy and life satisfaction are intended to serve as a chain-mediated effect between physical exercise and achievement motivation.

Demographic variables (e.g., gender, grade, major, residence) have always been of great interest in previous studies on achievement motivation (Khumalo et al., 2011). In terms of current related research, the majority of survey participants are primarily college students and community members. Therefore, this study will focus specifically on college students as the target population.

In sum, we have established a mediation hypothesis model by considering physical exercise as the independent variable, achievement motivation as the dependent variable, and self-efficacy and life satisfaction as mediating variables. Within this framework, we examined multiple mechanisms through which physical exercise influences achievement motivation, specifically focusing on the mediating roles of self-efficacy and life satisfaction. Our goal was to contribute to a long-term, multi-dimensional plan for improving achievement motivation. In this regard, we aim to inspire university administrators to develop well-grounded theoretical management frameworks that enhance the achievement motivation of college students. Additionally, we seek to provide practical insights for fostering future career achievement motivation among college students. We posited the following hypothesis: (1) Physical exercise can have a positive impact on college students’ achievement motivation. (2) Self-efficacy can serve as a mediating factor between engagement in sports activities and motivation for academic achievement among college students. (3) Life satisfaction can mediate between physical exercise and college achievement motivation. (4) Self-efficacy and life satisfaction can serve as a chain-mediated role between physical exercise and college students’ achievement motivation.

## 2 Materials and methods

### 2.1 Participants and procedure

The survey was conducted using a random sampling approach, with students from Southwest University in Chongqing being selected as the study participants. Prior to the commencement of the study, we obtained informed consent from college students after contacting school administrators. For freshmen, sophomores, and juniors, electronic questionnaires were distributed through physical education teachers on a class-by-class basis and completed on-site. Seniors were randomly selected for participation. A total of 900 female college students agreed to take part in our study. On average, participants took approximately 10–20 min to complete the questionnaire at normal completion rates. After excluding missing data and invalid questionnaires, the final sample consisted of 844 (93.78%) individuals primarily aged between 18 and 24 years (M = 20.32, SD = 1.67).

The survey data for this study was collected anonymously, and informed consent forms were obtained from all participants. Following the completion of the survey, the research team provided them with corresponding 2 RMB souvenirs as a gesture of gratitude. This survey protocol has been approved by the Ethics Committee of Southwest University Human and adheres to the Helsinki Declaration of Ethical Standards.

### 2.2 Materials

#### 2.2.1 Physical exercise

We employed the revised Physical Activity Rating Scale-3 (PARS-3) to evaluate levels of physical exercise, as this method has demonstrated robust reliability and validity within the Chinese academic setting (Liang, 1994; Lin et al., 2022). Moreover, Javalle employs a motion scale to quantitatively assess the engagement in physical activities and the extent of physical exercise across diverse age cohorts, thereby enhancing the precision and reliability of questionnaire dissemination, thus substantiating the scientific efficacy of this scale (Javelle et al., 2023). This scale assesses college students’ engagement in physical activities across three dimensions: intensity, duration, and frequency. Each indicator is rated on a 5-point scale (1–5), and the physical exercise score is calculated as follows: Exercise Volume Score = Intensity Score × (Duration Score - 1) × Frequency Score. Scores range from 0 to 100, with scores ≤19 indicating low exercise levels, scores between 20 and 42 indicating moderate exercise levels, and scores ≥43 denoting high exercise levels. Higher scores correspond to greater degrees of physical activity. In our study, the scale exhibited excellent internal consistency and construct validity with a Cronbach’s *α* coefficient of 0.848.

#### 2.2.2 Self-efficacy

The Chinese version of the General Self-Efficacy Scale (GSES) was used to assess individuals’ levels of self-efficacy (Zhang and Schwarzer, 1995), which represents their subjective evaluation of confidence in performing specific behaviors (e.g., “I can always solve problems if I try hard enough.”). Consisting of 10 items, the GSES utilizes a 4-point Likert scale ranging from 1 (strongly disagree) to 4 (strongly agree), with higher scores indicating greater self-efficacy. In this study, the questionnaire exhibited high internal consistency with a Cronbach’s *α* coefficient of 0.929.

#### 2.2.3 Life satisfaction

The Satisfaction with Life Scale (SWLS), developed by Pavot and Diener (1993), was utilized in this study to assess individuals’ overall life satisfaction. This scale consists of 5 items that are rated on a 7-point Likert scale ranging from 1 (strongly disagree) to 7 (strongly agree). Higher scores indicate higher levels of life satisfaction. The SWLS demonstrates suitability for evaluating the satisfaction of Chinese university students and exhibits excellent reliability (Huo and Kong, 2013), as evidenced by a Cronbach’s *α* coefficient of 0.911.

#### 2.2.4 Achievement motivation

We employed the Achievement Motivation Scale (AMS) developed by Ye and Kunt (1992) to assess students’ level of achievement motivation, which encompasses two dimensions: striving for success and avoiding failure. The AMS comprises 30 items, rated on a 4-point Likert scale ranging from 1 (strongly disagree) to 4 (strongly agree). This scale has demonstrated robust reliability and validity among Chinese student populations (Gao et al., 2019), as evidenced by a Cronbach’s α coefficient of 0.968 obtained in this study.

### 2.3 Data analysis

Statistical analysis was conducted using SPSS 27.0. Firstly, descriptive statistics were used to depict the demographic characteristics of participants by presenting frequencies and percentages. Secondly, Cronbach’s α test was employed to assess the internal consistency and reliability of four scales. Pearson correlation analysis was then performed on the independent variable, dependent variable, and two mediator variables. Next, Harman’s single-factor test was implemented to evaluate common method biases in the variables. Regression analysis was carried out with demographic factors as predictor variables and four outcome variables to explore their predictive relationships with each other. Finally, Model 6 in the SPSS Process program was utilized for mediation effects analysis to further investigate physical exercise’s predictive role on college students’ achievement motivation while revealing a chain-mediated effect involving self-efficacy and life satisfaction in physical exercise and achievement motivation. A sample size of 5,000 obtained through autonomous sampling methods ensured robust standard errors and a 95% bias-corrected confidence interval (CI). If CI does not include zero, it indicates significant mediation effects (DiCiccio and Efron, 1996).

## 3 Results

### 3.1 Common method bias tests

Given that the data in this study was collected through questionnaires and the survey participants were homogeneous, it is possible that a common method bias exists. To address this concern, Harman’s single-factor test was employed to assess the presence of such biases (Podsakoff et al., 2003). Upon conducting the analysis, it was found that the first factor accounted for 31.377% of variance, which falls below the critical threshold of 40% (Zhou and Long, 2004). This outcome confirms that there is no significant evidence of common method bias within this study.

### 3.2 Descriptive analysis

Demographic variables, including gender, age, grade level, major, and academic year, have consistently garnered attention in prior research on enhancing achievement motivation (Aguayo et al., 2019). Gender has been a focal point due to the lack of consensus in academia regarding whether gender-related differences exist in college students’ achievement motivation. Consequently, before examining the mediating role of self-efficacy and life satisfaction, descriptive statistics were performed on the collected demographic data, specifically focusing on gender, grade level, major, and place of residence (Table 1). In the survey sample, a total of 844 participants were included, comprising 398 males and 446 females. Specifically, the distribution by academic year was as follows: 222 freshmen, 238 sophomores, 185 juniors, and 199 seniors. Additionally, 439 participants were from social sciences, while 405 were from natural sciences. Furthermore, 452 participants were from urban areas, and 392 were from rural or town areas.

**Table 1:** Demographic characteristics.

Variable	Level	*N*	Percentage
Gender	Male	398	47.16
	Female	446	52.84
Grade	Freshmen	222	26.3
	Sophomores	238	28.2
	Juniors	185	21.92
	Seniors	199	23.58
Degree subject	Social sciences major	439	52.01
	Natural sciences major	405	47.99
Place of residence	City	452	53.55
	Villages	392	46.45

### 3.3 The relationships between physical exercise, self-efficacy, life satisfaction, and achievement motivation

The Pearson correlation analysis was employed in this section to investigate the relationship between four variables: physical exercise, self-efficacy, life satisfaction, and achievement motivation. As shown in Table 2, a significant correlation (*p* < 0.01) was observed among all variables. Specifically, physical exercise exhibited positive correlations with self-efficacy, life satisfaction, and achievement motivation. Moreover, there was a significant positive association between self-efficacy and life satisfaction. Additionally, a strong positive correlation existed between self-efficacy and achievement motivation. Overall findings suggest that college students with higher levels of physical exercise, self-efficacy, and life satisfaction tend to exhibit elevated scores on the scale measuring achievement motivation. These results reflect robust levels of achievement motivation among individuals while also indicating the beneficial impact of physical exercise on one’s level of achievement motivation.

**Table 2:** Correlation coefficients between the study variables.

Variables	M	SD	1	2	3	4
1. Physical exercise	3.09	1.03	1			
2. Self-efficacy	2.21	0.77	0.288**	1		
3. Life satisfaction	4.61	1.58	0.333**	0.439**	1	
4. Achievement motivation	2.32	0.72	0.464**	0.506**	0.399**	1

**p < 0.01.

The relationship between variables was further investigated by conducting a collinearity diagnostic experiment among college students to examine the association between physical exercise, self-efficacy, and life satisfaction. The results indicated that the VIF values for physical exercise, self-efficacy, and life satisfaction were below 5 (1.157, 1.275, 1.314), while the tolerances exceeded 0.3 (0.864, 0.785, 0.761). Consequently, no issues of collinearity were observed and subsequent tests can be pursued.

### 3.4 Explicit regression analysis of variables in the chain mediation model

The data is found to conform to a normal distribution, as indicated by the skewness (range: −0.627 to 0.427) and kurtosis (range: −1.092 to −0.668), both of which fall within the acceptable range of less than 2 and 7, respectively, (Kline, 2015). The subsequent analysis involved conducting four linear regression analyses, with the four variables in the model serving as outcome variables (as presented in Table 3). Demographic factors and other variables were utilized as predictor variables. The findings revealed that: (1) When PARS-3 was the outcome variable, gender (*β* = 0.098, *p* < 0.01), grade (β = 0.123, *p* < 0.01) were positive predictors. (2) When GSES was the outcome variable, only grade (β = 0.150, *p* < 0.01) served as a positive predictor. (3) When SWLS was the outcome variable, gender (β = 0.089, *p* < 0.01), grade(β = 0.135, *p* < 0.01) were positive predictors. (4) When AMS was the outcome variable, gender (β = 0.069, *p* < 0.05), grade (β = 0.144, *p* < 0.01) were positive predictors.

**Table 3:** Regression analysis of variables in the chain mediation model.

Outcome variable	Predictor variable	R^2^	F	β	SE	*t*	*p*
PARS-3	Gender	0.025	6.411	0.098	0.070	2.860	<0.01
	Grade			0.123	0.031	3.622	<0.01
	Degree subject			0.054	0.070	1.578	0.115
	Place of residence			−0.029	0.071	−0.857	0.392
GSES	Gender	0.020	5.193	0.039	0.052	1.130	0.259
	Grade			0.150	0.023	4.396	<0.01
	Degree subject			−0.002	0.052	−0.050	0.960
	Place of residence			0.014	0.053	0.409	0.683
SWLS	Gender	0.023	5.941	0.089	0.108	2.588	<0.01
	Grade			0.135	0.048	3.970	<0.01
	Degree subject			0.027	0.108	0.781	0.435
	Place of residence			−0.007	0.108	−0.213	0.832
AMS	Gender	0.022	5.631	0.069	0.050	2.026	<0.05
	Grade			0.144	0.022	4.230	<0.01
	Degree subject			0.016	0.049	0.469	0.639
	Place of residence			0.006	0.050	0.167	0.867

### 3.5 Mediated effect test: the effects of physical exercise on achievement motivation

**Figure 1:**
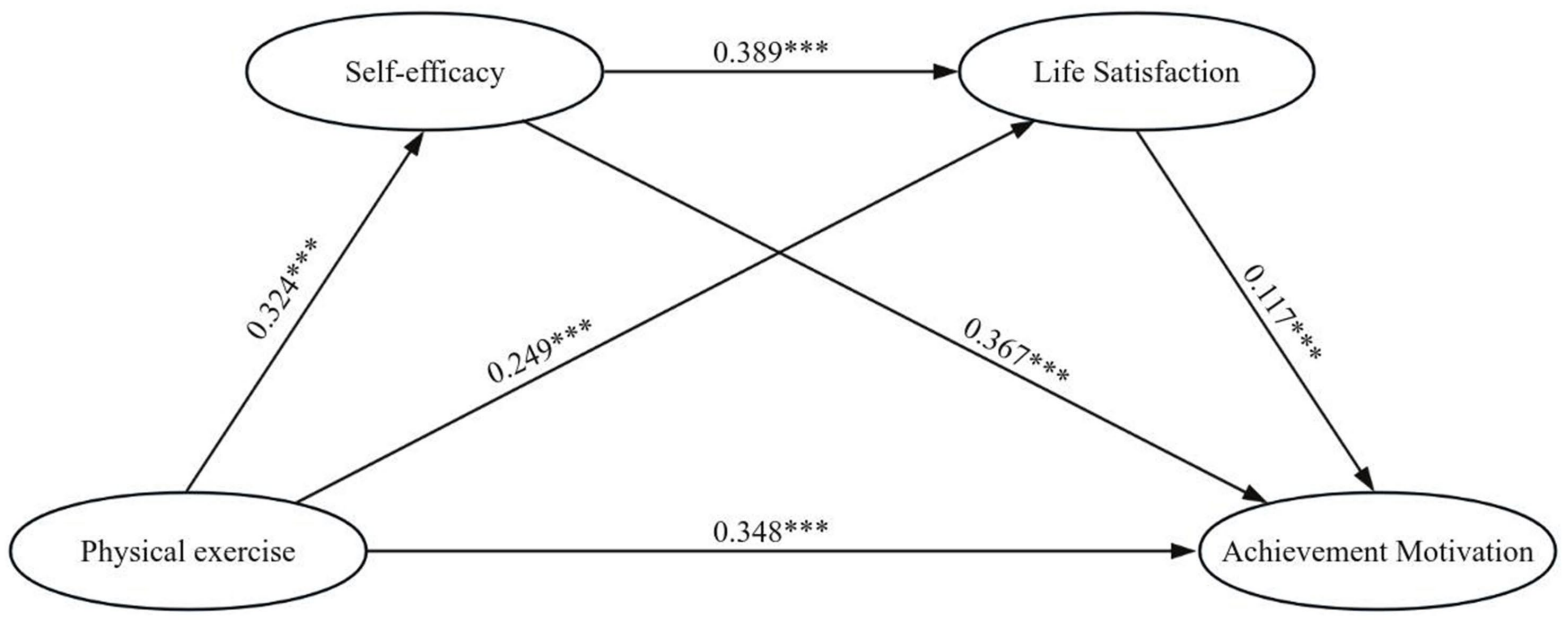
Model showing the mediating roles of self-efficacy and life satisfaction in the relationship between physical exercise and achievement motivation.

Based on the aforementioned assumption, physical exercise is considered an independent variable while achievement motivation is regarded as a dependent variable. Additionally, self-efficacy and life satisfaction are treated as mediating variables. To examine the mediating effects, we establish a chain mediation model using SPSS Process 3.5 plugin model 6 and conduct bootstrap tests for both the overall sample and different coping strategies. The results indicate that physical exercise exerts a significant direct effect on achievement motivation. Additionally, physical exercise has a total mediating effect of 0.163 on achievement motivation through the pathways of self-efficacy and life satisfaction, which accounts for 32.02% of the overall effect (0.512) of physical exercise on achievement motivation. The mediation effect included three indirect pathways: physical exercise→self-efficacy→achievement motivation (effect value 0.119), physical exercise→life satisfaction→achievement motivation (effect value 0.029), and physical exercise→self-efficacy→life satisfaction→achievement motivation (effect value 0.015). Based on these findings from sample analysis, we derive a mediation model diagram (Figure 1). Importantly, all path coefficients in this study are statistically significant at a certain level (see Table 4).

**Table 4:** Analysis of direct effects of physical exercise on achievement motivation and mediation effects of self-efficacy and life satisfaction.

	Effect size	Boot SE	LLCI	UUCI	Relative mediation effect
Total mediating effect	0.512	0.027	0.459	0.566	
Direct effect	0.348	0.031	0.284	0.406	67.98%
Total indirect effect	0.163	0.029	0.108	0.236	32.02%
Indirect 1	0.119	0.018	0.087	0.161	23.27%
Indirect 2	0.029	0.009	0.014	0.050	5.76%
Indirect 3	0.015	0.004	0.007	0.025	2.99%

In summary, the findings demonstrate a significant chain-mediated effect of self-efficacy and life satisfaction in linking physical exercise to achievement motivation. This confirms the direct impact of physical exercise on levels of achievement motivation and highlights its potential to enhance life satisfaction by influencing individuals’ sense of self-efficacy, thereby positively affecting achievement motivation.

## 4 Discussion

This study investigated the mechanistic role of physical exercise in achievement motivation among college students, using self-efficacy and life satisfaction as mediating factors to construct an intermediate model.

Firstly, the research findings based on the utilization of a more intuitive and concise PARS-3 scale demonstrate that physical exercise has a direct and positive predictive effect on college students’ level of achievement motivation. Engaging in sports activities not only enhances their capacity for success-driven motivation but also aligns with previous research results (Lou et al., 2023), thereby supporting H1. Moreover, consistent with prior conclusions, active participation in physical exercise not only mitigates negative emotions experienced by college students but also fosters psychological resilience through the pleasurable experience of sports, ultimately cultivating a positive drive towards achieving motivation (Anderson et al., 2006). Further demographic analysis reveals that gender and grade differences have significant impacts on individual achievement motivation. The gender disparity may be attributed to the fact that male college students are more likely to identify suitable adjustment points in sports and utilize interpersonal communication methods, thus effectively alleviating stress and enhancing levels of achievement motivation (Tremblay et al., 2023). Conversely, grade disparities may stem from varying degrees of accumulated anxiety caused by academic burdens (Schladitz et al., 2024). Furthermore, given that all students are within the university campus environment, the influence of their major and place of residence on achievement motivation appears to be less significant. Therefore, it indicates that when examining the impact of sports exercise on college students’ achievement motivation, careful consideration must be given to various demographic factors. It is crucial to prioritize individual differences in the development of sports exercise plans in order to select rational and scientifically-backed sports activities, exercise intensity, frequency, and duration for maximizing the enhancement of achievement motivation. For instance, college students can incorporate periodic low-intensity running exercises into their routine as an effective means of fat and calorie consumption, thereby bolstering their self-control and willpower. This approach also represents a vital self-regulatory framework essential for maintaining optimal levels of achievement motivation (Xu et al., 2018).

In addition to the direct impact, we also examined the indirect influence of physical exercise on achievement motivation. Our findings support H2 by demonstrating that personal self-efficacy acts as a mediator between physical exercise and achievement motivation. Physical exercise not only releases hormones like dopamine (Berse et al., 2015; Toselli and Spiga, 2017), which contribute to the development of a more optimal physique for college students and enhance their body satisfaction and self-efficacy, but it also promotes the adoption of advantageous strategies among individuals with high self-efficacy (Ouyang et al., 2020). This indicates that they approach challenges with a positive mindset towards achieving success (Chen et al., 2022). Therefore, physical exercise can influence achievement motivation through its effect on self-efficacy levels. Given the significant role played by self-efficacy in mediating the relationship between physical exercise and achievement motivation, targeted interventions should be implemented on campus, such as offering specialized counseling courses and providing specific cognitive plans aimed at enhancing students’ goal aspirations aligned with their abilities. These interventions will consistently stimulate achievement motivation through successful task completion.

According to the findings from indirect pathway 2, there is a specific indirect impact of physical exercise on achievement motivation mediated by life satisfaction. This suggests that physical exercise not only directly predicts achievement motivation but also indirectly enhances it through the mediating role of life satisfaction. These results are consistent with previous studies (Antunes et al., 2022; Rodrigues et al., 2023), thereby supporting hypothesis H3. Prior research has demonstrated that physical exercise can enhance hippocampal plasticity and reduce brain inflammation, leading to a reduction in negative emotional interference and an inclination towards more rational interpretations and attributions of events, ultimately maximizing life satisfaction and pleasure (De Miguel et al., 2021). Moreover, the enhancement in life satisfaction contributes to an increase in gray matter volume within the right hemisphere of the brain, resulting in clearer and more reliable cognitive abilities. Consequently, this provides college students with broader perspectives and more convenient approaches when encountering relevant challenges (Kong et al., 2014; Erickson et al., 2015). Considering the points mentioned above, it is crucial for society to establish a comprehensive nationwide fitness program, improve the monitoring system for student life satisfaction, organize significant sporting events and inclusive group activities with the goal of cultivating a sense of social belonging among college students. These actions will effectively alleviate feelings of loneliness and decrease the likelihood of depression while promoting positive motivation towards academic achievements (Samadieh and Rezaei, 2024). Educational institutions should allocate resources strategically to sustain motivation levels by establishing counseling services and workshops that facilitate students’ experience of fulfillment within campus life under the guidance and companionship provided by teachers and peers (Lardier et al., 2020). Simultaneously, families ought to promote gentle communication methods such as engaging in outdoor activities like hiking, jogging or participating in sports with their children as means to alleviate academic fatigue experienced by college students. This approach will foster a healthy and harmonious family environment that enhances overall familial happiness while concurrently promoting steady improvements in achievement motivation (Xue et al., 2024).

Ultimately, this study demonstrates that physical exercise has a positive influence on achievement motivation through the chain-mediated effects of self-efficacy and life satisfaction. Thus, hypothesis H4 is supported. Although there is limited direct evidence for the multidimensional relationship between physical exercise, self-efficacy, life satisfaction, and achievement motivation, previous studies have already confirmed the role of physical exercise in fostering an optimistic mindset (Blanchette and Richards, 2010). This optimism helps college students maintain rational thinking when perceiving and evaluating current events, thereby enhancing their sense of self-efficacy and enabling them to overcome apprehension towards the unknown while responding positively and adaptively to uncertain future circumstances (Yu and Song, 2022). Moreover, individuals with high levels of self-efficacy tend to possess resilience and a forward-looking sense of purpose which enhances overall cognitive openness leading to increased life satisfaction and happiness (Liu et al., 2024). Consequently, this fosters positive emotions among individuals and empowers them to fully utilize their potential in promoting achievement motivation within the college student population as a whole group (Deng et al., 2024). To effectively cultivate college students’ achievement motivation, educators should prioritize nurturing students’ self-efficacy while also creating a supportive living environment conducive to enhancing student satisfaction. Furthermore, educational institutions should guide students in developing regular exercise habits so they can reap the benefits associated with sports participation including improved physical health and mental well-being as well as enhanced social relationships and strengthened self-identity; all serving as solid foundations for future achievements motivations.

## 5 Limitations and prospects

Although our research has contributed to a deeper understanding of the relationship between physical exercise and achievement motivation among college students in academic circles, there are certain limitations that need to be acknowledged. Firstly, the research sample was limited to college students in Chongqing city, which may limit the generalizability of the findings. The cultural background, educational resources, and social environment in different regions exert varying influences on the achievement motivation of college students. Consequently, future research should consider incorporating cross-regional and cross-cultural research designs, taking into account factors such as the level of regional development and the richness of cultural heritage, to enhance the external validity of the findings. Secondly, this study primarily relied on questionnaire surveys for data collection. While this method offers convenience for operational purposes and quantitative analysis, it may not fully capture students’ underlying psychological states accurately or their sports performance. For instance, questionnaires might not precisely reflect students’ thoughts and emotions in specific situations. To enrich the depth and richness of future research endeavors, qualitative methods such as conducting in-depth interviews with students or employing comparative case studies could be integrated to obtain more comprehensive data samples. Lastly, this study may lack depth in its investigation scope. Future studies could consider assessing a wider range of demographic factors to effectively validate the effectiveness of intervention measures employed within these contexts effectively. Given the complexity and diversity inherent in college student life experiences, it is crucial for future research efforts to explore additional variables and factors while developing a more comprehensive model that facilitates an enhanced understanding of positive relationships promoting achievement motivation (e.g., family background, study situation, life pressure).

## 6 Conclusion

The present study investigates the relationship between physical exercise, self-efficacy, life satisfaction, and achievement motivation among college students. It demonstrates the positive impact of physical exercise on achievement motivation and confirms the independent mediating role of self-efficacy and life satisfaction in the association between physical exercise and achievement motivation. We propose that guiding college students to engage in appropriate physical exercise can significantly enhance their self-efficacy and life satisfaction, thereby fostering positive achievement motivation when they face challenges. Incorporating structured physical exercise programs into institutional policies would not only underscore the importance of physical activity for educators but also better promote the physical health and motivational levels of college students.

## Data Availability

The raw data supporting the conclusions of this article will be made available by the authors, without undue reservation.
